# Evaluation of a recombinant insect-derived amylase performance in simultaneous saccharification and fermentation process with industrial yeasts

**DOI:** 10.1007/s00253-015-7098-8

**Published:** 2015-11-07

**Authors:** Ewelina Celińska, Monika Borkowska, Wojciech Białas

**Affiliations:** Department of Biotechnology and Food Microbiology, Poznań University of Life Sciences, ul. Wojska Polskiego 48, 60-627 Poznań, Poland

**Keywords:** Amylase, Yeast, Heterologous expression, Raw starch, Simultaneous saccharification and fermentation, Bioethanol

## Abstract

Starch is the dominant feedstock consumed for the bioethanol production, accounting for 60 % of its global production. Considering the significant contribution of bioethanol to the global fuel market, any improvement in its major operating technologies is economically very attractive. It was estimated that up to 40 % of the final ethanol unit price is derived from the energy input required for the substrate pre-treatment. Application of raw starch hydrolyzing enzymes (RSHE), combined with operation of the process according to a simultaneous saccharification and fermentation (SSF) strategy, constitutes the most promising solutions to the current technologies limitations. In this study, we expressed the novel RSHE derived from an insect in *Saccharomyces cerevisiae* strain dedicated for the protein overexpression. Afterwards, the enzyme performance was assessed in SSF process conducted by industrial ethanologenic or thermotolerant yeast species. Comparison of the insect-derived RSHE preparation with commercially available amylolytic RSH preparation was conducted. Our results demonstrate that the recombinant alpha-amylase from rice weevil can be efficiently expressed and secreted with its native signal peptide in *S. cerevisiae* INVSc-pYES2-*Amy1* expression system (accounting for nearly 72 % of the strain’s secretome). Application of the recombinant enzyme-based preparation in SSF process secured sufficient amylolytic activity for the yeast cell propagation and ethanol formation from raw starch. (Oligo)saccharide profiles generated by the compared preparations differed with respect to homogeneity of the sugar mixtures. Concomitantly, as demonstrated by a kinetic model developed in this study, the kinetic parameters describing activity of the compared preparations were different.

## Introduction

Bioethanol still appears as the most attractive “green alternative” to depleting fossil fuels (Bai et al. 2008) and compares favorably with 2,3-butanediol or 1-butanol, for example, with respect to production capacity and technology advancement. Microbe-derived ethanol can be produced from sucrose, starch, or lignocellulosic biomass, which are the most commonly used feedstocks. Bioethanol production from sugar crops (sugarcane and sugar beet) account for about 40 % of the total bioethanol production, and nearly 60 % correspond to starchy material conversion (Biofuels Platform 2010). Starch is the dominant feedstock consumed for bioethanol production in North America and Europe, due to their agro-ecological conditions. The principal advantage of starchy biomass utilization for bioethanol production lies primarily in simple and efficient technologies (Lee 2007; van Zyl et al. 2012). Considering the high contribution of bioethanol to the global fuel market, any improvement in its major operating technologies is economically very attractive (Bai et al. 2008). According to suitable calculations, the production cost of ethanol is primarily derived from the consumption of raw materials and energy input (Bai et al. 2008; McAloon et al. 2000; Wang et al. 2007). Along with the final product purification and distiller’s dried grains with solubles (DDGS) drying, the highest energy input in starch-based ethanol technology is consumed for mashing step, involving cooking of the substrate at high temperatures (90–95 °C) to gelatinize starch. Thus, reduction of the energy required for the high-temperature mashing step was indicated as an important factor to be solved in starch-to-ethanol technologies (Sánchez and Cardona 2008).

Raw starch hydrolyzing enzymes (RSHE) constitute an excellent solution to this limitation. Application of RSHE, instead of traditional enzymes, allow to exclude the costly high-temperature mashing step from the bioethanol production pipeline, which in the further perspective may lead to more sustainable processing (Białas et al. 2014). Conventional enzymatic preparations used in traditional bioethanol production processes contain liquefying alpha-amylase and saccharifying glucoamylase, operating at 90 to 110 °C and 60 to 70 °C, respectively (Sharma et al. 2007). RSHE preparation constitutes a mixture of alpha-amylase and glucoamylase, jointly conducting hydrolysis of noncooked starch directly into fermentable sugars at lower temperature (30 to 48 °C). Hence, application of RSHE eliminates the necessity for the cost-intensive cooking step and therefore reduces the energy input per unit of produced bioethanol. According to the results by Robertson et al. (2006), the reduction in the overall energy input, conveyed by the usage of RSHE in ethanol production, equals to 10–20 %. A direct consequence of RSH enzyme application in the bioethanol production pipeline is running the process according to a simultaneous saccharification and fermentation (SSF) strategy (Białas et al. 2014). Simultaneous provision of amylolytic agent (RSHE - acting on nonpretreated substrate) and yeast cells (consuming saccharides released from starch granules) offers several advantages over the traditional two-step bioethanol production technologies (separate saccharification and fermentation). First of all, end-product inhibition of amylolytic enzymes is avoided, as the products of catalysis are continuously consumed by the yeast cells; thus, starch decomposition kinetics is improved. Second of all, the number of reactors needed in the production pipeline is reduced, contributing to reduction in the investment costs. Finally, in the combined RSHE-SSF process, further reduction in the operational costs is conveyed by application of RSH enzymes.

*Saccharomyces* spp. are still the first choice fermentative strains in the biotechnological production of ethanol. A wide array of traits make some particular strains belonging to this genus perfectly suited for the ethanol production processes, such as good fermentative capacity, high tolerance to ethanol and other inhibitors, and ability to grow rapidly under anaerobic conditions, which are typically established in large-scale fermentation vessels (Mussatto et al. 2010). Nevertheless, other yeast species are tested in bioethanol production technologies to verify their performance. As demonstrated in a number of reports, some nonconventional yeast species have been found to exhibit unusual tolerance to stresses encountered during bioethanol production, e.g., *Zygosaccharomyces rouxii* (osmotolerance—important in very high gravity mash fermentation), *Kluyveromyces marxianus* and *Ogataea* (*Hansenula*) *polymorpha* (thermotolerance—valuable in SSF processes), *Dekkera bruxellensis* (ethanol tolerance), *Pichia kudriavzevii* (furan derivatives tolerance—valuable in lignocellulosic ethanol technologies), and *Zygosaccharomyces bailii* (acetic acid tolerance) (recently reviewed by Radecka et al. 2015). Thermotolerance is a particularly valuable trait for SSF strategy, as it allows to compromise the temperature optima of the enzyme (≥40 °C) and the yeast cells (30 °C) operation. Thus, testing thremotolerant ethanol producer performance in RSHE-SSF systems appears to be a reasonable approach towards optimization of the process. Ethanol production represents one of the pivotal fields of *K. marxianus* strains exploitation (Raimondi et al. 2013).

In this study, we have cloned and expressed an insect (*Sitophilus oryzae*) alpha-amylase in the *Saccharomyces cerevisiae* INVSc1-pYES2 system, dedicated for protein overexpression, with the aim to produce highly active enzymatic preparation to be tested in an SSF process. *S. cerevisiae* is continuously used as a cell factory for the recombinant protein production, taking advantage of its robust growth capacity, ease of genetic manipulations with a wide array of genetic engineering tools available, low production costs, and feasibility of scaling-up of the process. Both biopharmaceutical proteins as well as industrially relevant enzymes have been produced in this species (Mattanovich et al. 2012). As we have previously shown, *S. oryzae*-derived alpha-amylase is able to decompose raw starch granules of various plant origins (Celińska et al. 2015a). This should make it particularly useful in combined RSHE-SSF processes. To examine the enzyme performance in the RSHE-SSF process, we expressed the gene in the INVSc1-pYES2 expression system, set up batch bioreactor cultures to produce the enzyme in larger quantities, purified it via FPLC technique, and finally tested the enzyme performance in the RSHE-SSF system, in comparison with commercially available RSHE preparation. In the SSF processes, two wild-type yeast species were used: typical ethanologenic industrial yeast strain, *S. cerevisiae* Ethanol Red, and thermotolerant *K. marxianus* DSMZ 5422. The results of the insect-derived alpha-amylase production in INVSc1-pYES2 bioreactor cultures together with analysis of secretion efficiency, as well as *S. cerevisiae* and *K. marxianus* strains performance in RSHE-SSF cultures with a commercial RSHE and the insect-derived-based RSH enzymatic preparation, are all presented in this report. Kinetic modeling of the enzymatic preparations action on raw starch granules was also performed and presented in this study.

## Materials and methods

### Strains and small-scale flask cultivations

All the strains used in this study are listed in (Table 1). The INVSc1 strain was routinely maintained in YPD medium (g/L): yeast extract, 10; bactopeptone, 20; glucose, 20; and agar, 20. Before any experiments, the strain’s *ura*-phenotype was verified in SC(−/+)U medium (g/L): glucose, 20; yeast nitrogen base without amino acids, 6.7; yeast synthetic drop-out medium supplement without uracil, 1.4; uracil, 76 mg; and agar, 20. In SC-U _induction medium, glucose was exchanged to raffinose (7 g/L), and galactose was provided as an inducer (14 g/L). *E. coli* JM109 strain was used for the pYES2(+/−*Amy1*) vectors construction and propagation. *E. coli* JM109 and all the derivatives were cultured in LB medium (g/L): yeast extract, 5; bactopeptone, 10; NaCl, 10; and agar, 15, supplemented with ampicillin (100 mg/L), when required. Flask cultivations for molecular biology protocols were carried out in 300-mL nonbaffled Erlenmayer flasks, with culture volumes of 50–100 mL, on a rotary shaker, at 250 rpm, under aerobic conditions at 30 or 37 °C for *S. cerevisiae* and *E. coli*, respectively.

**Table 1:** Strains, vectors, oligonucleotides and enzymes used in this study

Name	Characteristics	Application	Supplier/reference
Strains			
*Saccharomyces cerevisiae* INVSc1	*Genotype: MATa his3Δ1 leu2 trp1-289 ura3-52/MATα his3Δ1 leu2 trp1-289 ura3-52; Phenotype: His–, Leu–, Trp–, Ura–*	Host for expression of the recombinant alpha-amylase	Life Technologies, Invitrogen [Carlsbad, CA, USA].
*Escherichia coli* JM109	*F’[traD36,proAB+,lacIq,Δ[lacZ]M15], endA1,recA1,hsdR17[rk-,mk+], mcrA,supE44,λ-,gyrA96,relA1, Δ[lacproAB],thi-1*	Host for routine cloning, vector propagation, assembly of a complete vector	Sigma-Aldrich [USA]
*Kluyveromyces marxianus DSMZ 5422*	*Wild-type industrial strain*	Simultaneous saccharification and fermentation	DSMZ German Collection of Microorganisms and Cell Cultures [Germany]
*Saccharomyces cerevisiae Ethanol Red*	*Wild type industrial strain, dedicated for the ethanol industry, characterized by high ethanol tolerance, fast acting strain*	Simultaneous saccharification and fermentation	Lesaffre [France]
Vectors			
pYES2	http://tools.lifetechnologies.com/content/sfs/manuals/pyes2_man.pdf	expression vector used for transformation of *S. cerevisiae* INVSc1 strain	Life Technologies, Invitrogen [Carlsbad, CA, USA]
pGEM-T-Easy	https://pl.promega.com/resources/protocols/technical-manuals/0/pgem-t-and-pgem-t-easy-vector-systems-protocol/	subcloning of the *Amy1* gene and DNA fragments composing pYLTEF vector, sequencing	Promega Co., [USA]
Oligonucleotides			
AMY_*Hin*dIII_F	AAGCTTAACAAAATGTCCAAGGTGCTCGCCCTGC	*Amy1* gene amplification in pYES2 vector	this study
AMY_*Xba*I_R	AATCTAGACTAGTGGTGGTGGTGGTGGTGC		
Enzymes		Activity acc. to Producer	
Stargen001^TM^	A mixture of granular starch hydrolyzing enzymes: *Aspergillus kawachi* alpha-amylase (generates dp1-dp6) and *Aspergillus niger* glucoamylase (generates dp1) temp. 48–50 °C, pH 4.0–4.5	>456 GSHU/g (GSHU = granular starch hydrolyzing units)	Genecor International, Palo Alto, CA, USA
SpritaseGA 14400 L	Glucoamylase from *Aspergillus niger* (generates dp1) Temp. 55–60 °C, pH 3–6, opt. pH 4.5	>5000 u/g	Enzym, Poland (Novozymes cooperator)

### Development of a pYES2-Amy1 DNA construct

Standard molecular biology techniques were used throughout this study (Sambrook and Russell 2001). The cDNA sequence, encoding alpha-amylase (*Amy1*) gene from *S. oryzae* (gb|HQ158012.1) was codon-optimized for expression in yeast species, at GenScript Inc. (Piscataway, USA) as described in our previous report (Celińska et al. 2015a). Codon-optimized sequence (gb|KP027641) was 100 % identical in a primary amino acid structure with the original sequence from *S. oryzae*. Vectors and oligonucleotides used in this study are summarized in (Table 1). Restriction enzymes, shrimp-alkaline phosphatase, and DNA molecular markers for electrophoresis were purchased from Thermo Fisher Scientific Inc. (Walthman, MA, USA). DNA T4 ligase was obtained from New England Biolabs (UK). Plasmid DNA Isolation Kit and DNA Fragments Purification Kit (Gel Out kit) were purchased from A&A Biotechnology (Gdynia, Poland). DNA Taq polymerase was purchased from Qiagen (Germany). Amplification of *Amy1* gene was set up in a Veriti^®^ ThermalCycler (Applied Biosystems), using *AMY_Hin*dIII*_F* and *AMY_Xba*I*_R* primer pair (0.5 μM each) and approx. 20 ng of DNA template, in a final volume of 25 μL, using the following temperature profile: 94 °C for 5 min, (94 °C for 30 s, 56 °C for 30 s, 72 °C for 90 s) × 25, and 72 °C for 3 min. The obtained *Amy1* amplicon was cloned in a pGEM-T-Easy vector (Promega Co., USA) and verified through sequencing (Genomed sequencing facility, Poland). The *Amy1* gene was cloned in a *Hin*dIII/*Xba*I-digested pYES2 vector, after *Hin*dIII/*Xba*I excision from pGEM-T-Easy.

### *S. cerevisiae* transformation and selection of positive clones

Preparation of *S. cerevisiae* INVSc1 competent cells and transformation with the pYES2-*Amy1* construct were completed according to the protocol supplied by the manufacturer of the pYES2 system. The *ura + Amy1 +* prototroph phenotype was verified in YPS medium (g/L): yeast extract, 10; bactopeptone, 20; glucose, 20; agar, 20; and soluble starch, 10. After 24 h growth, the biomass was scraped and 5 % of iodine solution (I_2_ in KI) was poured onto the plate to visualize translucent zones. INVSc1 parental strain was used as a negative control.

### Protein extract preparation and the amylase activity assay

Protein extracts were prepared by resuspending the cellular pellets in breaking buffer (0.1 M sodium phosphate buffer, 5 μM DTT, 1 mM PMSF, 5 % glycerol) with glass beads (Sigma Aldrich Co., USA) and disruption of the cells by repeated cycles (5×) of mixing at 30 strokes/s for 1 min in a MixerMill MM400 (Retsch) and incubation on ice for 1 min. The cellular debris was then separated by centrifugation (24,652×*g*, 4 °C, 10 min). The protein concentration was determined according to the method described by Bradford (1976), using bovine serum albumin (BSA) as a standard. The amylase activity assays were all carried out according to the Nelson-Somogyi method (Nelson 1944) versus a standard curve prepared with glucose. The concentration of background sugars contained within the enzymatic preparations was each time assessed and taken into account in the calculations. All the OD 560-nm measurements were done in three technical replicates (Analytik Jena Spectrophotometer and WinASPEKT Software). One activity unit was defined as the amount of enzyme that released reducing sugar ends equivalent to 1 μmol of glucose per 1 min under the specified assay conditions.

### Bioreactor cultivations

Bioreactor cultivations were carried out in BIOSTAT^®^ A plus (Sartorius) stirred-tank bioreactors, with a total volume of 5 L and a culture medium volume of 1 L. The SC-U_induction medium was inoculated with 22-h-old INVSc1-*Amy1* strain’s biomass at the amount resulting in the final OD600 of 0.4. pH and temperature were adjusted to 5.5 and 30 °C throughout the process. Stirring and aeration were automatically adjusted to maintain oxygen saturation of the culture at 30 %, at an air flow of 2 vvm. Foam formation was controlled by automatic addition of AntiFoam 204 (Sigma-Aldrich). Biomass growth was monitored through optical density measurements at 600 nm wavelength. Concentration of raffinose and galactose was analyzed through high-performance liquid chromatography (HPLC), as described previously (Celińska et al. 2015b), and standards were purchased from Sigma-Aldrich (St. Louis, MO, USA). Amylolytic activity was determined in the cellular fraction and in the culture media. The activity assay was accompanied by SDS-PAGE analysis of the total protein from the respective fraction, according to a standard method (Laemmli 1970). The cultivations were carried out in four independent runs. Purification of the heterologous alpha-amylase from the culture medium was conducted according to previously described methodology (Celińska et al. 2015a). Briefly, extracellular proteins were precipitated with ammonium sulfate (to the final saturation of 80 %, at 4 °C) overnight and separated by centrifugation (4234×*g*, 4 °C, 45 min). The protein deposit was suspended in a binding buffer (phosphate buffer, 20 mM, pH 7.4; NaCl, 0.5 M; imidazole, 20 mM), filtered through a 0.45-μm syringe filter (Millex, Millipore), and loaded onto the ÄKTA FPLC system (ÄKTA Pharmacia GE FPLC) equipped with a HisTrap HP column (5 mL, GE Healthcare), with Ni^2+^ ions immobilized on sepharose. The purification procedure was carried out under increasing gradient of an elution buffer (phosphate buffer, 20 mM, pH 7.4; NaCl, 0.5 M; imidazole, 0.5 M). Fractions were immediately analyzed for the amylase activity according to the Nelson-Somogyi method (Nelson 1944). The purified enzymatic preparation was further used in SSF processes.

### Simultaneous saccharification and fermentation processes

#### Biological material

*K. marxianus* DSMZ 5422 and *S. cerevisiae* Ethanol Red are described in Table 1. Two amylolytic preparations were used in this experiment: (1) commercially available Stargen001™ preparation (Genecor International, Palo Alto, CA, USA) (hereafter referred to as Stargen); and (2) a mixture of the heterologous insect-derived alpha-amylase (ref. to as AMY), obtained in this study, and commercially available glucoamylase Spritase GA14400L (Enzym, Poland) (ref. to as GlucoAMY). The mixture is hereafter referred to as AMY + GlucoAMY. Characteristics of the commercial enzymes are described in (Table 1). The two enzymes (AMY and GlucoAMY) were included in the custom enzymatic preparation to provide activities required for complete starch degradation. AMY attacks starch granules, randomly hydrolyzing internal glycosidic bonds in the polymer, while GlucoAMY attacks terminal bonds from the nonreducing end of the polymer and dextrins generated by AMY. Appropriate counterparts of both activities are present in the Stargen preparation. To unify the amount of the amylolytic activity added into the SSF cultures, amylolytic activity of both preparations (Stargen and AMY + GlucoAMY) was determined according to the Nelson-Somogyi method, as described above.

#### SSF cultures conditions

The SSF cultures were carried out in 300-mL nonbaffled Erlenmayer flasks containing 50 mL of production medium composed of (g/L): native rice starch, 150; chloramphenicol, 100 mg; diammonium phosphate, 5; and microelements solution II according to Barth and Gaillardin (1996), 1 mL. The medium has not been sterilized, to avoid starch gelatinization. Initial pH was adjusted to 4.5. Glass marbles of approx. 5 mm in diameter were added into the flasks to facilitate mixing and suspension of the yeast biomass and the starch. The amylolytic preparations were added into the media at the following amounts: (1) 30 AU of Stargen preparation, (2) 15 AU of each insect-derived alpha-amylase (AMY) and glucoamylase (GlucoAMY). The SSF processes were initiated immediately after the enzyme provision, by inoculation of the production medium at 10 % with the yeast strains, pre-cultured in YPD medium (rotary shaker incubator at 30 °C, 250 rpm, for 24 h). Cellular density of the inocula was assessed at 120 × 10^8^ and 14 × 10^8^ cfu/mL for *K. marxianus* and *S. cerevisiae*, respectively. The SSF processes were conducted in a rotary shaker incubator, with shaking at 100 rpm and temperatures of 37 and 30 °C for *K. marxianus* and *S. cerevisiae*, respectively, for 146 h. Ethanol production and yeast cell viability were monitored throughout the experiment. The cultures were carried out in three biological replicates. Potential spontaneous decomposition of raw starch was monitored in blank cultures, where neither yeast cells nor enzymes were provided. Kinetics of the starch decomposition, as well as the profile of generated (oligo)saccharides in control cultures, supplemented with enzymes and lacking the yeast cells, was monitored through the HPLC technique (described hereafter).

#### Analytical procedures

##### Determination of ethanol concentration–gas chromatography (GC)

Ethanol concentration in the yeast-inoculated cultures was determined using the GC technique. Clear liquid supernatants were obtained through centrifugation at 24,652 × g, for 10 min, at 4 °C, and passed through a 0.45-μm syringe filter (Millex, Millipore). Prior to sample extraction, n-pentanol was added into the samples as an internal standard. Extraction was carried out according to the following protocol: 0.8 mL of the sample was mixed with equal amount of n-butanol, 0.2872 g of NaCl, and 25 μL of n-pentanol, vortexed for 5 min and incubated at room temperature to allow phases separation. The organic phase was transferred to chromatography vials and analyzed using Agilent Technologies 7890A GC apparatus, equipped in Zebron ZB-WAX column (30 m × 250 μm × 0.25 μm), under the following conditions: injector temperature 250 °C, oven program—1 min at 80 °C, followed by 20 °C/min at 120 °C, followed by 120 °C/min at 220 °C and 3 min at 220 °C; detector temperature 250 °C; and mobile phase—hydrogen 30 mL/min, air 300 mL/min, and helium 20 mL/min. Ethanol concentration was determined using previously prepared standard curve.

##### Determination of (oligo)saccharides concentration–HPLC

Concentration of saccharides of polymerization degrees from dp1 to dp7 was monitored through HPLC analysis in the control cultures without yeast provision. Clear liquid supernatants were obtained through centrifugation at 24,652×*g*, for 10 min, at 4 °C, and passed through a 0.45-μm syringe filter (Millex, Millipore). Agilent Technologies 1200 series chromatograph used in this analysis was equipped with Rezex RSO-Oligosaccharide Ag^+^ 200 × 10 mm (Phenomenex) column, autosampler G1329B, double pump G1312B, and refractic index detector G1362A. Ten microliters of the samples was loaded onto the column. H_2_O was used as the eluent at 0.3 mL/min, under isocratic conditions. The analysis was conducted at 80 °C. Quantitative and qualitative identification of the compounds was carried out using external standards and the peak height (automatic determination and integration using ChemStation for LC 3D systems, Agilent).

##### Living cell counts.

The yeast cell viability was monitored throughout the SSF processes. Collected samples were decimally diluted and subsequently plated onto two parallel YPD agar plates. After 24 h incubation at 30 °C, the colonies were counted.

### Comparison of the enzymatic preparations performance—kinetics modeling

Assumptions for the kinetics studies were as follows: (1) glucose (dp1), maltose (dp2), maltotriose (dp3), maltotetraose (dp4), maltopentaose (dp5), maltohexaose (dp6), and maltoheptaose (dp7) constitute the major end-products of the action of the alpha-amylases contained within the enzymatic preparations used; glucoamylases generate glucose (dp1). Action of alpha-amylases and glucoamylases in either Stargen or AMY + GlucoAMY preparations is considered a joint action of “an amylolytic activity.” Hence, glucose (dp1) concentration, being the final product of the “amylolytic activity” action, was taken into consideration in the calculations. (2) Starch granules subjected to enzymatic digestion constitute homogenous substrate. (3) In a course of enzymatic hydrolysis of starch, the rate of catalysis changes—in the first phase, the reaction is more rapid, as the concentration of the product is low and feedback inhibition is also low; in the second phase—the concentration of the product is high, increasing the feedback inhibition imposed on the “amylolytic activity” being examined.

The experimental results of starch decomposition by Stargen and AMY + GlucoAMY at 30 and 37 °C, measured by the concentration of released glucose, were subjected to nonlinear regression analysis using commercial software Statistica 12 (StatSoft, Inc.) and the numerical method of Levenberg–Marquardt (Moré 1977) for coefficient estimation. Fitting of the model with the experimental data was assessed by a value of the determination coefficient (*R*^2^).

Mathematical description of the starch hydrolysis reaction, expressed as glucose production, catalyzed by the enzymes contained in Stargen and AMY + GlucoAMY preparations, is provided by Eq. 1.

1 $$ Y=Y0+A\ast \left(1- \exp \left(-k1\ast t\right)\right)+B\ast \left(1- \exp \left(-k2\ast t\right)\right) $$

where k1 and k2 are the rate constants of glucose release into the reaction medium in the first phase and the second phase of the starch decomposition, respectively. According to the third assumption, k1 > k2. Y0 is the concentration of glucose at 0 h. A and B are model coefficients in g/L; *t* refers to the time of the reaction. Coefficients A, B, k1, and k2 were estimated based on Levenberg—Marquardt algorithm.

## Results

### Expression and secretion of an insect *Amy1* gene in *S. cerevisiae*-pYES2 expression system

The final pYES2-*Amy1* DNA construct was used for transformation of INVSc1 competent cells. Transformation efficiency equaled to 6.0 × 10^2^ transformants/μg of plasmid DNA. The obtained transformation efficiency is close to the efficiency guaranteed by the manufacturer of about 10^3^ colonies/μg of plasmid DNA. All the prototrophs growing on SC-U selective plates were able to produce an active form of the recombinant alpha-amylase, resulting in 100 % selectivity of the expression system (number of the amylase-producing strains per total number of the prototrophs).

### Production of the recombinant alpha-amylase in batch bioreactor cultures

**Fig. 1:**
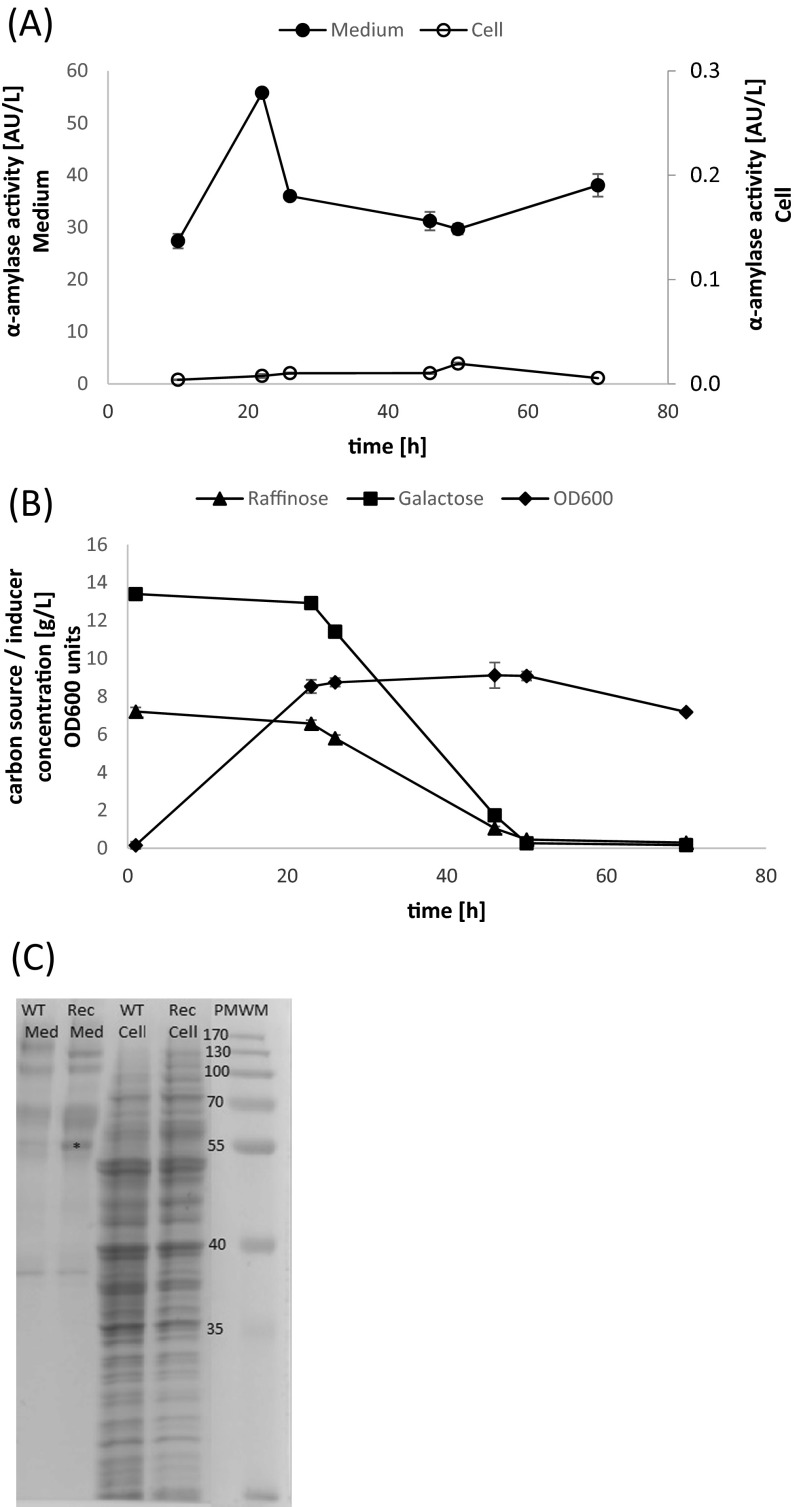
Batch bioreactor production of the alpha-amylase in *S. cerevisiae* INVSc1-pYES2-*Amy1*. **a** Time-course accumulation of the recombinant alpha-amylase in the medium and the cellular fraction, **b** carbon source and inducer concentration and biomass accumulation in a representative *S. cerevisiae* INVSc1-pYES2-*Amy1* batch bioreactor culture. Y axis: *closed circles*—alpha-amylase activity in the medium; *open circles*—alpha-amylase activity in the cell; *closed diamond*—biomass growth in OD600 units; *closed triangle*—raffinose concentration; *closed square*—galactose concentration. X axis: time of culturing in h. *Error bars* indicate ±SD. **c** SDS–PAGE electrophoretic separation of the total intracellular and extracellular protein fractions of the INVSc1 and the INVSc1-pYES2-*Amy1* strains. Proteins contained within the culture medium (Med), intracellular proteins (Cell), parental strain INVSc1 (WT), the recombinant strain (Rec—INVSc1-pYES2-*Amy1*). *Asterisk* (approx. 53 kDa) indicates the protein band, corresponding to the molecular weight of the recombinant amylase, expressed in the recombinant strain. *PMWM* protein molecular weight marker (PageRuler Prestained Protein Ladder, LifeTechnologies)

During the batch bioreactor cultures in SC-U induction medium, the main fraction (>99 %) of the recombinant alpha-amylase activity was detected in the culture medium, as demonstrated by both activity assay (Fig. 1a, Table 2) and SDS-PAGE electrophoresis (Fig. 1c). The amylolytic activity detected inside the cells remained at the negligible level throughout the culture. The peak in the recombinant alpha-amylase activity was observed at approx. 24 h of culturing (55.77 AU/L) (Fig. 1a). The extracellular enzyme production was the most rapid within the first 22 h of culturing (volumetric productivity of 2.53 ± 0.004 AU/(L*h)), when the concentration of the inducer was still high (Fig. 1b). Afterwards, the concentration of galactose started to decrease, and the volumetric productivity reached 0.54 ± 0.03 AU/(L*h) at the end of the cultivation (70 h). In this study, depending on the analyzed fraction, either intracellular or extracellular, the active alpha-amylase constituted up to approx. 72.58 % (specific activity of 0.62 AU/mg) of the total proteins in the medium and less than 0.05 % (0.007 AU/mg) in the cellular fraction (Table 2). The extracellular alpha-amylase was purified from the culture medium to apparent homogeneity. The purified enzymatic preparation of the recombinant alpha-amylase was further used in SSF processes.

**Table 2:** Kinetic parameters describing production of the recombinant alpha-amylase in *S. cerevisiae* INVSc-pYES2-*Amy1* batch bioreactor cultures

	% of total proteins [[mg/mL AMY^a^]/ [mg/mL total proteins]] [%]		% of AMY secreted [medium mg/ total mg] [%]	Volumetric productivity Medium [AU/[L*h]] ± SD
time h	medium	cell		
10	-	-	-	2.74 ± 0.14
22	72.58	0.017	99.98	2.53 ± 0.004
26	30.12	0.023	99.92	1.38 ± 0.002
46	7.76	0.024	99.69	0.68 ± 0.039
50	7.17	0.049	99.32	0.59 ± 0.02
70	6.99	0.019	99.72	0.54 ± 0.03

*% of total proteins* refers to the percentage of the alpha-amylase contained within the total amount of proteins, measured in the medium and in the cellular fractions. *% of AMY secreted* refers to the percentage of the alpha-amylase secreted. *Volumetric productivity Medium* refers to the volumetric productivity of the alpha-amylase, measured in the medium fraction

^a^mg/mL AMY was calculated based on the purified alpha-amylase activity. The enzyme was purified through affinity chromatography, to apparent homogeneity. 1 mg of the purified alpha-amylase contained 1.175 AU

### Simultaneous saccharification and fermentation processes

The SSF processes carried out in this study aimed at production of ethanol from native starch granules by two wild-type yeast strains, *K. marxianus* DSMZ5422 and *S. cerevisiae* Ethanol Red, using two mixtures of RSH enzymes, AMY + GlucoAMY and Stargen, at the temperatures optimal for the respective yeast species growth (37 or 30 °C).

**Fig. 2:**
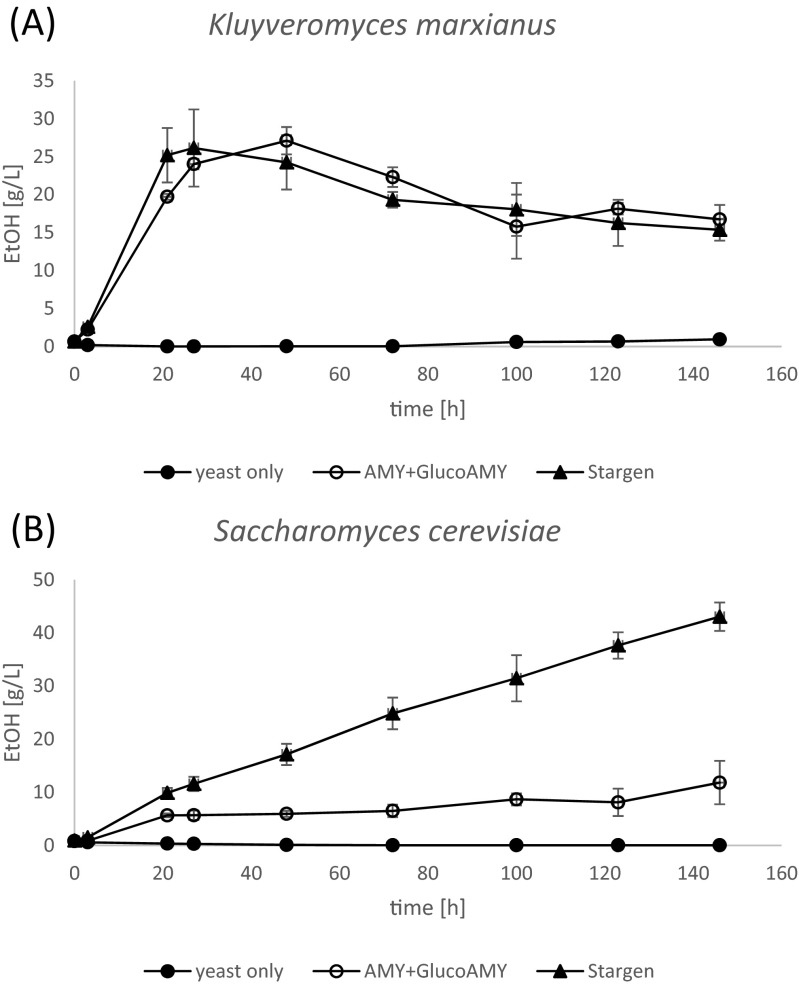
Ethanol production by *K. marxianus* DSMZ 5422 (**a**) and *S. cerevisiae* Ethanol Red (**b**) in the SSF processes. Y axis: ethanol concentration in g/L. X axis: time of the SSF process in h. *Closed circles*—SSF control culture variants in which only yeast cells were provided, without any amylolytic agent; *open circles*—SSF culture with AMY + GlucoAMY enzymatic preparation used as the amylolytic agent; *closed triangles*—SSF culture with Stargen enzymatic preparation used as the amylolytic agent. *Error bars* indicate ± SD

With respect to *K. marxianus* cultures (Fig. 2a), no significant difference in the ethanol production was observed depending on the type of enzymatic preparation present in the culture medium. In both cases, when either AMY + GlucoAMY or Stargen preparation was used as the amylolytic agent, the kinetics of ethanol production was similar, and the highest concentration reached approx. 27 g/L between 20 and 50 h of culturing (27.1 ± 1.81 g/L at 48 h with AMY + GlucoAMY vs. 26.15 ± 5.07 g/L at 27 h with Stargen). Volumetric productivity of ethanol synthesis in the initial phase of culturing (until 27 h) reached 0.89 ± 0.02 and 0.969 ± 0.188 g/(L*h) with AMY + GlucoAMY and Stargen, respectively. Continuation of culturing led to a decrease in the ethanol concentration (to 16.73 ± 1.9 and 15.37 ± 1.44 g/L with AMY + GlucoAMY and Stargen, respectively), resulting in the final volumetric productivity of approx. 0.1 g/(L*h) for both variants. No ethanol was detected in *K. marxianus* cultures, when no external RSH enzyme preparation was provided, indicating lack of sufficient native amylolytic activity by the strain. Some minimal liquefied-starch-decomposing activity was however observed in YPS agar plates, visualized by Lugol iodine staining (data not shown).

In the case of *S. cerevisiae* SSF cultures (Fig. 2b), the kinetics of ethanol production differed significantly between Stargen and AMY + GlucoAMY preparation-containing cultures. With the former enzymatic preparation, ethanol was accumulated rapidly and throughout the whole process duration, at high and relatively constant volumetric productivity 0.505 ± 0.09 and 0.295 ± 0.02 g/(L*h) at 3 and 146 h of culturing, respectively. The final ethanol concentration reached 43.03 ± 2.66 g/L at 146 h. On the other hand, when AMY + GlucoAMY preparation was provided as the amylolytic agent, ethanol was synthetized slowly, at relatively low and decreasing volumetric productivity (0.29 ± 0.049 and 0.08 ± 0.028 g/(L*h) at 3 and 146 h, respectively), reaching the final concentration of 11.81 ± 4.08 g/L. As in the case of *K. marxianus*, no ethanol was formed by *S. cerevisiae* cells, when amylolytic preparations were not provided.

**Fig. 3:**
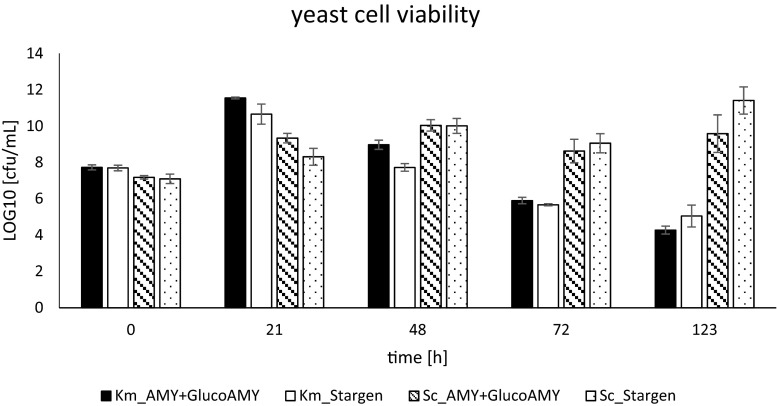
Yeast cell viability of *K. marxianus* DSMZ 5422 and *S. cerevisiae* Ethanol Red strains during SSF processes. Y axis: living cell counts in cfu/mL in logarithmic scale. X axis: time points in the SSF process in h. *Black*: Km_AMY + GlucoAMY: *K. marxianus* culture supplemented with AMY + GlucoAMY preparation; *White*: Km_Stargen: *K. marxianus* culture supplemented with Stargen preparation; *Striped*: Sc_AMY + GlucoAMY: *S. cerevisiae* culture supplemented with AMY + GlucoAMY preparation; *Dotted*: *S. cerevisiae* culture supplemented with Stargen preparation. *Error bars* indicate ± SD

The yeast cell viability was monitored throughout the SSF cultures. As presented in Fig. 3, the general trend of *K. marxianus* cell propagation and viability was similar when either AMY + GlucoAMY or Stargen was used as the amylolytic agent. High biomass propagation at the initial 21 h of culturing was followed by a gradual decrease in the living cell counts, proceeding until the end of culturing. On the other hand, living cell counts of *S. cerevisiae* strain remained relatively stable (10^8^ to 10^10^ cfu/mL) after the initial biomass propagation.

**Fig. 4:**
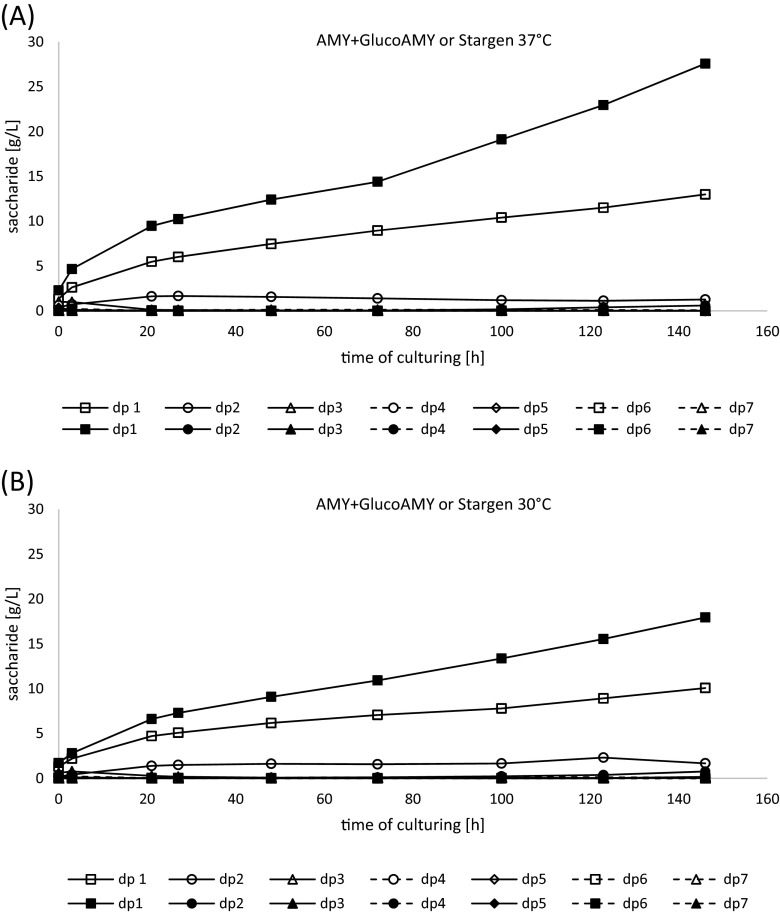
(Oligo)saccharide composition profile generated during SSF process with raw starch treated with either AMY + GlucoAMY (*open symbols*) or Stargen (*closed symbols*) preparations at 37 °C (**a**) or 30 °C (**b**). The profiles were determined in the control cultures without yeast cells provision. Additional blank cultures, containing neither yeast cells nor enzymatic preparations, were conducted to assess the extent of spontaneous decomposition of raw starch—not shown. Y axis: concentration of (oligo)saccharide of polymerization degree of dp1 to dp7 in g/L in the SSF process containing **a** AMY + GlucoAMY (*open symbols*) and Stargen (*closed symbols*) at 37 °C, **b** AMY + GlucoAMY (*open symbols*) and Stargen (*closed symbols*) at 30 °C. X axis: time of the SSF process in h.

To monitor the kinetics of starch decomposition by the two compared enzymatic preparations, concentration of (oligo)saccharides from dp1 to dp7 was determined in blank cultures (without yeast cells) in the course of culturing (Fig. 4a, b and Table 3). The extent of potential spontaneous decomposition of starch triggered by prolonged mixing and elevated temperature was monitored in blank cultures (neither yeast nor enzymes provided); only negligible amounts of saccharides were detected at the end of incubation. As it can be observed in Fig. 4a, b as well as Table 3, Stargen and AMY + GlucoAMY enzyme preparations generated a slightly different profile of saccharides. While the saccharide profile generated by Stargen was represented nearly solely by glucose (dp1) (97.65 % at 37 °C and 95.14 % at 30 °C), treatment with the latter enzymatic preparation generated a mixture of saccharides, although dominated by dp1 (90.37 % at 37 °C and 82.81 % at 30 °C), but with other saccharides present. The overall amount of sugars released from raw starch was higher for Stargen preparation under both temperature conditions (28.23 vs. 14.38 g/L and 18.86 vs. 12.17 g/L, at 37 and 30 °C, respectively), when compared with AMY + GlucoAMY preparation.

**Table 3:** (Oligo)saccharide profiles generated in the SSF processes with either GlucoAMY + AMY or Stargen amylolytic preparations acting on raw starch granules under 30 or 37 °C

T [°C]	Enzyme	Unit	dp1	dp2	dp3	dp4	dp5	dp6	dp7
37 °C	GlucoAMY + AMY	[%]	90.37	8.83	0.23	nd	nd	nd	0.57
		[g/L]	12.99	1.39					
	Stargen	[%]	97.65	2.11	0.07	nd	nd	nd	0.17
		[g/L]	27.57	0.66					
30 °C	GlucoAMY + AMY	[%]	82.81	13.75	1.34	nd	1.0	nd	1.1
		[g/L]	10.1	2.1					
	Stargen	[%]	95.14	4.05	0.12	nd	0.46	nd	0.23
		[g/L]	17.94	0.92					

The results are provided either in % of the overall amount of detected saccharides, or in g/L, as detected through HPLC

*Nd* not detected

**Fig. 5:**
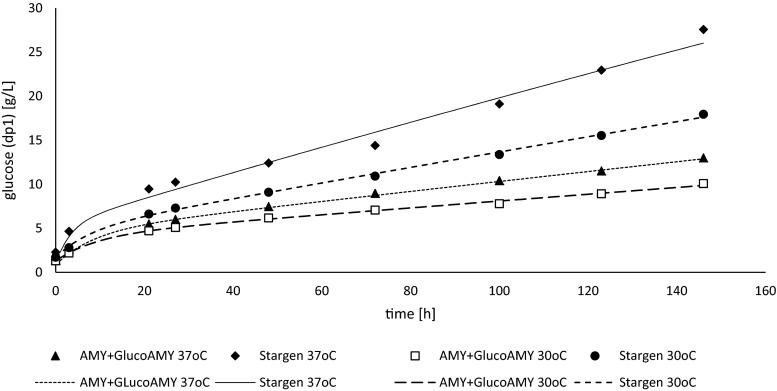
Fitting of the experimental results of glucose release during raw starch hydrolysis by AMY + GlucoAMY and Stargen enzymatic preparations at 37 and 30 °C to the values predicted by the model equation (Eq. 1) and corresponding coefficients (Table 4). *Lines* represent predicted values and *symbols* experimental values. Y axis: concentration of glucose (dp1) in g/L. X axis: time of the starch hydrolysis in h. Starch hydrolysis and the resultant glucose release performed by: AMY+GlucoAMY preparation at 37 °C (*closed triangles* and *small dashed line*); Stargen preparation at 37 °C (*closed diamonds* and *continuous line*); AMY+GlucoAMY preparation at 30 °C (*open squares* and *wide dashed lines*); Stargen preparation at 30 °C (*closed circles* and *medium dashed lines*)

To compare the catalytic activity of the two enzymatic preparations under investigation towards raw starch granules, we have applied nonlinear regression analysis and Levenberg–Marquardt algorithm for coefficient estimation. The estimated coefficients corresponding to the two-term equation (Eq. 1) are provided in (Table 4). The model fitting to the experimental data is given by the value of determination coefficient *R*^2^ ((Table 4) and is also schematically presented in (Fig. 5)).

**Table 4:** Coefficients of a raw starch hydrolysis kinetic model

Enzyme/temperature	Y0 [g/L]	A [g/L]	k1 [/h]	k2 [/h]	B [g/L]	*R* ^2^
AMY + GlucoAMY 37 °C	1.343	3.307	0.118	0.00035	166.5	0.992
Stargen 37 °C	2.289	3.255	0.304	0.000905	163.9	0.987
AMY + GlucoAMY 30 °C	1.301	2.872	0.108	0.000238	165.8	0.993
Stargen 30 °C	1.708	3.02	0.133	0.000551	166.9	0.995

The kinetic model coefficients corresponding to Eq. 1, estimated using the numerical method of Levenberg–Marquardt. Y0 is the initial glucose concentration at 0 h of SFF processes; A and B are model coefficients; k1 and k2 are the rate constants of glucose release into the reaction medium in the first and the second phase of the starch decomposition, respectively; *R* ^2^ is the determination coefficient assessing accuracy of the model fitting to the experimental results. The first column indicates the enzymatic preparation used and the reaction temperature

## Discussion

The ultimate aim of this study was to assess the performance of an insect-derived RSH enzyme in the SSF process of raw starch-to-bioethanol production. During the first phase of this study, the enzyme was produced in a laboratory *S. cerevisiae* INVSc1 strain, dedicated for protein overexpression.

*S. cerevisiae* is a well-established expression platform for the production of heterologous proteins, including commercialized therapeutic polypeptides. A variety of starch-decomposing enzymes originating from bacteria (*Streptococcus bovis*, *Bacillus amyloliquefaciens*, or *B. stearothermophilus*) or fungal species (*Debaryomyces occidentalis*, *Aspergillus awamori*, or *Lipomyces kononenkoae*) have been expressed in either laboratory or industrial *S. cerevisiae* strains (e.g., Khaw et al. 2006; Kim et al. 2010; Steyn and Pretorius 1991; Steyn et al. 1996; Shigechi et al. 2002). To the best of our knowledge, no report on the expression of an insect-derived amylolytic enzyme in *S. cerevisiae* has been published, to date. A significant proportion of the studies on the expression of amylolytic genes in *S. cerevisiae* fall into a strategy aiming at developing a consolidated biocatalyst (or consolidated bioprocessing—CBP), able to efficiently decompose starch and to produce ethanol simultaneously. A combination of two major characteristics of this species, namely high capacity for ethanol production, together with exploitation as a well-established protein expression platform, has emerged an idea of engineering *S. cerevisiae* for direct conversion of starch to ethanol (recently reviewed in (Görgens et al. 2014)). Although CBP appears as one of the most attractive solutions to the current limitations of biotechnological ethanol production, the other strategies cannot be neglected, especially that the currently operating technologies of bioethanol production rely on the separate addition of an amylolytic agent and a fermentative microorganism. Hence, our approach relied on different assumptions. First, we used *S. cerevisiae* INVSc1 laboratory strain, dedicated for protein overexpression, as a cell factory for the enzyme production. The INVSc1-pYES2 system used in this study is claimed to be an expression platform for an efficient, inducible expression of recombinant proteins (primary reports on GAL promoter exploited in this systems: Giniger et al. 1985; West et al. 1984). Afterwards, we used the obtained enzymatic preparation in the RSHE-SSF process with an industrial ethanologenic strain *S. cerevisiae* Ethanol Red strain or, alternatively, with a wild-type thermotolerant *K. marxianus* DSMZ5422 strain, to take advantage of the best qualities of the respective yeasts.

According to the results presented in Fig. 1a, c and Table 2, the major fraction of the alpha-amylase produced in the bioreactor cultures of *S. cerevisiae* INVSc-pYES2-*Amy1* was located in the culture medium, indicating efficient secretion of the heterologous protein. Signal sequences are the key factors controlling protein secretion. Native *S. cerevisiae* leader sequences, in addition to foreign and synthetic leader sequences, have been successfully used to target heterologous proteins for secretion (Hou et al. 2012). The pYES2 vector used in this study is not equipped with any signal peptide-encoding element that could contribute to the expressed protein secretion. Thus, it appeared that the native signal peptide from *S. oryzae* of 17 AA residues, as predicted by the PrediSI tool (http://www.predisi.de/), is operable in the secretory pathway of the *S. cerevisiae* cells. The typical signal peptide, belonging to “Sec-type” peptides, most common for *S. cerevisiae* secretome, consist of three subsequent domains (Yang et al. 2006): (1) N-domain containing at least one arginine or lysine (positively charged) residue; (2) H-domain composed of amino acids that easily form into α-helical conformation in the membrane during translocation, i.e., a stretch of 11–14 hydrophobic residues (helix-breaking residues such as glycine or proline are located at the end of this domain, being found to facilitate cleavage by specific signal peptidases); and (3) C-domain consisting of a specific signal peptidase cleavage site, composed of a consensus sequence A–X–A (X—any AA residue). It appeared that the signal peptide native for the rice weevil alpha-amylase (M–K–V L A L L V T V C F S V–A S A) conforms to most of the consensus *S. cerevisiae* signal peptide characteristics.

The kinetics of the heterologous alpha-amylase production in *S. cerevisiae* INVSc-pYES2-*Amy1* expression systems conforms to the typical batch production of recombinant proteins. The highest expression level (expressed in either AU/L or AU/(L*h)) was observed at the first 24 h of culturing (Fig. 1a, Table 2). Therefore, from a practical point of view, this process could be terminated after 24 h of culturing, as the culture prolongation did not bring any improvement in the recombinant alpha-amylase production. Comparable initial volumetric productivity of 2.17 ± 0.09 AU/(L*h) (vs. 2.53 ± 0.004 AU/(L*h) in this study) has been reported in our previous study, where the same gene was expressed in the *Yarrowia lipolytica* Po1g-pYLSC-*Amy1* expression system (Celińska et al. 2015a). A corresponding time-production profile has been observed by (Cardillo et al. 2008), expressing an enzyme-encoding gene in the same expression system INVSc-pYES2. More rapid production of chitinase in the same *S. cerevisiae*-pYES2 expression system was achieved by (Loc et al. 2013), where the enzyme activity increased continuously from 4 to 12 h (reaching 12 AU/L) and decreased rapidly afterwards.

Accumulation of the heterologous proteins in *S. cerevisiae* cells varies widely depending on the foreign gene being expressed. The average yields of the recombinant proteins produced in *S. cerevisiae* cells reach a maximum level of 1–6 % of the total intracellular protein (Mendoza-Vega et al. 1994). Heterologous alpha-amylase from *Sch. occidentalis* accounted for 12 % of the total secretome of the recombinant *S. cerevisiae* (Wang et al. 1998). In this study, the recombinant alpha-amylase accounted for up to 72 % of the recombinant strain’s secretome. However, when expressed in AU/L, the amount of the active recombinant alpha-amylase contained within the culture medium was observed to be lower when compared to our previous results (55.77 vs. 81 AU/L; in Celińska et al. 2015a). *Y. lipolytica*, which served as a host in that study, is known for having a highly efficient co-translational secretory pathway, which could potentially contribute to higher expression level of the secreted protein. The subsequent slight decrease in the extracellular alpha-amylase activity, observed in this study, can be attributed to either sequestration of a fraction of the enzyme in a persistent foam layer, making the enzyme unavailable for the activity assay (Clarkson et al. 1999) or lysis of the cell, suffering from sugar substrate shortage (Fig. 1b).

Purified enzymatic preparation of the heterologous alpha-amylase was further used in the SSF processes with wild-type yeast strains, *S. cerevisiae* Ethanol Red and *K. marxianus* DSMZ5422, which constituted the second phase of this study. *S. cerevisiae* Ethanol Red strain chosen for this experiment is a typical industrial yeast strain, characterized by a high ethanol-producing capacity and resistance to its elevated concentrations. *K. marxianus* DSMZ 5422 was chosen as a representative of thermotolerant yeast strains, which are particularly desired in SSF processes, allowing to compromise thermal optima of the RSHE and the yeast ethanol producer. The here applied set of enzymes operates best at the following temperatures: Stargen 48–50 °C, Spritase 55–60 °C (GlucoAMY), and alpha-amylase (AMY) according to preliminary studies, reported in (Celińska et al. 2015a), at 40 °C. Thermal conditions applied in this study in SSF processes had to be balanced between the optimal temperatures of the enzymes and the yeast cell metabolic activity/growth. Thus, for *S. cerevisiae*, we applied the highest temperature (30 °C) from the conventional temperature range used during in vitro cultivations (25 to 30 °C), which is also optimal for alcoholic fermentations (Torija et al. 2003). *K. marxianus* was reported to produce ethanol at the temperatures above 40 °C and to have a maximum growth temperature of 47 °C (Anderson et al. 1986), 49 °C (Hughes et al. 1984), or even 52 °C (Banat et al. 1992). However, our earlier experience, as well as the literature data (Raimondi et al. 2013), indicate that the temperature levels between 30 °C to a maximum of 40 °C are the most favorable for *K. marxianus* growth and ethanol production. Hence, the temperature of 37 °C was applied in SSF cultures with *K. marxianus*. The results presented in Fig. 4a, b and Table 3 indicate that both enzymatic preparations generated less saccharides at 30 °C, when compared to catalysis at 37 °C. The difference was even more clearly marked for Stargen enzyme (28.23 vs. 18.86 g/L), which produced 33.19 % less of the total saccharides (dp1–dp7) at the lower temperature. On the other hand, the total saccharide yield by AMY + GlucoAMY preparation (14.38 vs. 12.2 g/L) was 15.16 % lower at 30 °C when compared with 37 °C, suggesting its lower susceptibility to decreased temperature, in the analyzed range.

Based on the experimental results of starch hydrolysis by the compared enzymatic preparations, the kinetic model describing these reactions was proposed (Eq. 1) (corresponding coefficients are presented in (Table 4)). The proposed model describes the enzyme action kinetics with adequate adjustment, as denoted by high determination coefficient *R*^2^ values (for all conditions ∼0.99) (Table 4 and Fig. 5). According to the primary assumptions made for the kinetic studies, this model assumes action of only one “amylolytic activity,” comprising synergetic action of alpha-amylase and glucoamylase, generating glucose (dp1) as the final product. Such an approach has been earlier applied in similar studies (Białas et al. 2014; Davis 2008; Kroumov et al. 2006). Equation 1 comprises two terms representing respectively the first and the second phase of the starch decomposition reaction (as explained in the third assumption of the model description in the “Materials and methods” section). As it was previously demonstrated, a clear division of the starch enzymatic hydrolysis into the two phases (of low and high product concentration) is reasonable due to susceptibility of the catalysts to feedback inhibition by the product. According to Białas et al. (2014), amylolytic activities contained in the Stargen preparation are greatly inhibited by glucose, at negligible inhibition by starch, even though the cultivations were conducted according to the SSF strategy. Similar conclusions were drawn by Polakovic and Bryjak (2004), as well as by Kroumov et al. (2006). The ultimate aim of the kinetic studies carried out in this experiment was the quantitative comparison of catalytic activity of the two enzymatic preparations towards raw starch granules. The rate constants of the glucose release in both the first and the second phases of starch hydrolysis indicate that Stargen preparation performed better under the applied experimental conditions, as denoted by the ratio of the rate constant (k) values (2.58-fold higher k1 and 2.59-fold higher k2 at 37 °C; 1.23-fold higher k1 and 2.31-fold higher k2 at 30 °C). The k parameter comparison confirmed the preference of the enzymes contained in Stargen preparation for higher temperatures (as known from the enzyme specification provided by the manufacturer, as well as previous studies (e.g., Wang et al. 2007)), which is not beneficial for *S. cerevisiae* Ethanol Red strain. At the lower temperature (30 °C), the difference in the two preparations performance was slightly lower, especially in the first phase of the reaction, when the concentration of saccharides in the SSF process environment was low. Application of higher temperatures (of up to 40 °C), being more beneficial for the enzymes action, was possible only with selected thermotolerant *S. cerevisiae* strains (Hu et al. 2012).

The RSHE-SSF processes carried out in this study aimed at production of bioethanol. The results of ethanol production in the respective variants of this experiment are presented in Fig. 2a, b. Noteworthy, the data concerning (oligo)saccharide production in control flasks (Fig. 4a, b) cannot be directly extrapolated to the SSF cultures with ethanologenic yeast cells (Fig. 2a, b), since the kinetics of saccharide production could differ significantly, due to simultaneous consumption of generated saccharides by metabolically active yeast cell population.

For *K. marxianus* the SSF cultures were carried out at 37 °C. Under these conditions, Stargen preparation generated more saccharides, as presented in Fig. 4a. Nevertheless, it was not reflected by higher ethanol production, since irrespective of the amylolytic agent used, the kinetics of ethanol production was not significantly different (Fig. 2a). The most rapid ethanol accumulation was observed at the first 24 h of culturing. Afterwards, the production reached plateau. The most probable reason contributing to the observed phenomenon was toxic concentration (>20 g/L) of ethanol in the culture medium. *K. marxianus* is known to be low-ethanol tolerant (Rosa and Sá-Correia 1992). Relatively low tolerance to ethanol was correlated with the activity of the plasma membrane ATPase (Rosa and Sá-Correia 1992), being an intrinsic trait of a strain. As indicated in Fig. 3, *K. marxianus* viable cell population started to diminish after 24 h of culturing, which was concomitant with increased ethanol concentration above 26 g/L. A relatively large decrease in the ethanol concentration, proceeding from 48 h until the end of culturing, was observed in *K. marxianus* SSF cultures (Fig. 2a). The identified factors that could contribute to this observation were the following: (1) evaporation of the product from the heated and shaken flasks during prolonged incubation at lack of its production, and (2) consumption of ethanol for production of ethyl acetate. Ethyl acetate is a valuable aroma compound of sweet, fruity odor, widely applied as a food additive. *K. marxianus* is known to be a good producer of ethyl acetate (e.g., Löser et al. 2014 and 2015; Morrissey et al. 2015). In the medium supernatants, some small quantities of ethyl acetate in *K. marxianus* SSF cultures were detected (∼0.5 g/L) during GC analysis. But most probably, a significant proportion of this volatile compound could evaporate from the flasks, protected with cotton wool plugs solely, as the characteristic ethyl acetate odor was very intensive in an incubator. The maximum ethanol concentration of ∼27 g/L was reached between 24 to 48 h of culturing, with the peak point depending on the amylolytic agent used. Corresponding results (27.88 g/L of ethanol) were obtained in the SSF process with *K. marxianus* growing in pre-treated sunflower biomass (sulfuric acid at 121 °C) (Camargo et al. 2014). Maximum ethanol concentration of 19 g/L was obtained using *K. marxianus* CECT 10875 strain in the SSF process with pre-treated lignocellulosic materials (Ballesteros et al. 2004). When cheese whey permeate was used as a substrate for *K. marxianus* UFV-3, 76 and 80 g/L of ethanol were produced under oxygen-limited and anaerobic conditions (Silveira et al. 2005). Banat et al. (1996) cultured *K. marxianus* IMB3 strain in anaerobic chemostat fermentation (at 45 °C and dilution rate of 0.15 /h) and obtained 18 g/L of the final ethanol concentration. Modification of culturing mode (two-stage fermentation in sequence: one aerobic and one anaerobic; or two-stage anaerobic fermentation with cell recycling) allowed to increase the ethanol yield to 43 g/L at 45 °C and 77 g/L at 40 °C; these results also show that the elevated temperature (>40 °C) contributes to the decrease in the obtained ethanol yields in *K. marxianus* cultures.

*S. cerevisiae* Ethanol Red SSF cultures were conducted at 30 °C, at which the profiles of released (oligo)saccharides differed to a higher extent depending on the amylolytic agent provided (Fig. 4b) than at 37 °C. While application of Stargen preparation resulted merely in glucose (dp1) buildup, provision of AMY + GlucoAMY lead to accumulation of glucose (dp1), but also maltose (dp2), accounting for 13 % of the total saccharides released and analyzed in this study. The most probable reason contributing to this observation is the lower activity of the glucoamylase counterpart in the custom enzymatic preparation at lower temperature. A corresponding profile of saccharides, generated by Stargen, was observed by Wang et al. (2007). In that study, three types of amylolytic preparations (Stargen and two other commercially available conventional liquefying and saccharifying preparations) were compared. It was observed that Stargen is characterized by a distinctively homogenous profile of generated saccharides, represented nearly solely by glucose. Other types of preparations generated more heterogeneous profiles of (oligo)saccharides, in line with what was observed in this study. Both the profile of generated (oligo)saccharides as well as the differences in the kinetics of the amylolytic enzymes (measured by the rate constant k) could potentially contribute to some extent to the observed variations in the ethanol buildup. As presented in Fig. 2b, production of ethanol by *S. cerevisiae* Ethanol Red strain was ultimately 4-fold higher when Stargen preparation was used as the amylolytic agent, in comparison with AMY + GlucoAMY application (43.03 ± 2.66 vs. 11.8 ± 4.08 g/L). The final ethanol concentration in an experimental system Ethanol Red-Stargen was 4-fold higher than Ethanol Red-AMY + GlucoAMY but also nearly 3-fold higher than the ethanol concentration reached in any of the *K. marxianus*-based systems. The obvious reason accounting to this observation is the higher resistance of the industrial, ethanologenic strain to elevated levels of ethanol, as demonstrated by the results of yeast cell viability (Fig. 3). In Fig. 3, it can be seen that the living cell population of *S. cerevisiae* Ethanol Red strain was not altered after initial propagation during the SSF process, irrespective of the ethanol concentration. Even a slight increase in cfu/mL was observed at 123 h of culturing in Ethanol Red-Stargen SSF cultures, indicating a high metabolic activity of the yeast cells at this distal time-point, in contrast to *K. marxianus* cultures.

In the aforementioned study by Wang et al. (2007), no significant difference in the ethanol yield or its production rate was observed between SSF process variants, differing in the type of amylolytic agent used (thus in the generated saccharides profile). Correspondingly, all the kinetic parameters describing the SSF process were not statistically different. This contradicts our assumption that variation in the (oligo)saccharides profile generated by the enzymatic preparations could influence the ethanol production kinetics. In the study by Wang et al. (2007), it has been stated that lower concentration of sugars and their slower release rates are beneficial for the yeast strain’s performance during ethanol fermentation. However, due to prior liquefaction applied in that study (even for RSHE, Stargen), the concentration of accumulated sugars was significantly higher than in our experiments (up to nearly 200 g/L). Therefore, increased osmotic pressure might have unfavorably influenced the cell growth in that experiment. In this study, the SSF process was not preceded by the liquefaction step and any sugars released from the raw starch granules were instantly consumed by the growing yeast cells. Thus, a higher and more rapid sugar release by Stargen preparation (17.94 vs. 10.07 g/L of dp1 at 30 °C) was found to be favorable for the overall process efficiency, in contrast to what has been observed by Wang et al. (2007).

In conclusion, this study shows that the recombinant alpha-amylase from rice weevil can be efficiently expressed and secreted with its native signal peptide in the *S. cerevisiae* INVSc-pYES2-*Amy1* expression system. To the best of our knowledge, this is the first report on expressing insect amylolytic enzyme in *S. cerevisiae*. Evaluation of the enzyme performance in the SSF processes demonstrated that the insect amylase-based preparation, in a mixture with commercial glucoamylase, was useful as an amylolytic agent in the processes of raw starch-to-ethanol production by wild-type ethanologenic yeasts. Application of the AMY + GlucoAMY preparation provided sufficient amylolytic activity for the yeast cell propagation and ethanol formation. However, AMY + GlucoAMY preparation was characterized by less rapid decomposition of raw starch, when compared with the commercial Stargen preparation. (Oligo)saccharide profiles generated by the compared preparations differed with respect to homogeneity of the sugar mixtures. Concomitantly, as demonstrated by a kinetic model developed in this study, the kinetic parameters describing activity of the compared preparations differed. While in SSF processes with *K. marxianus* the type of amylolytic agent used had no significant influence on the final ethanol yield, the experimental system comprising *S. cerevisiae* Ethanol Red-Stargen performed significantly more efficient than the alternative *S. cerevisiae* Ethanol Red-AMY + GlucoAMY, in this regard.
